# Sidelobe Suppression with Resolution Maintenance for SAR Images via Sparse Representation

**DOI:** 10.3390/s18051589

**Published:** 2018-05-16

**Authors:** Xiaoxiang Zhu, Feng He, Fan Ye, Zhen Dong, Manqing Wu

**Affiliations:** 1 College of Electronic Science, National University of Defense Technology, No. 109 De Ya Road, Changsha 410073, China; xiaoxiang.z@yahoo.com (X.Z.); yefan311@sina.com (F.Y.); dongzhen@nudt.edu.cn (Z.D.); 2 China Electronic Technology Group Corporation (CETC), Wan Shou Road, Beijing 100000, China; wumanqing@ustc.edu.cn

**Keywords:** peak sidelobe ratio (PSLR), integrated sidelobe ratio (ISLR), amplitude error (AE), phase error (PE), sidelobe suppression, resolution maintenance

## Abstract

Severe sidelobe interference is one of the major problems with traditional Synthetic Aperture Radar (SAR) imaging. In the observation scene of sea areas, the number of targets in the observation scene is so small that targets can be regarded as sparse. Taking this into account, a method of sidelobe suppression, on the basis of sparsity constraint regularization, is proposed to reduce sidelobes of Gaofen-3 (GF-3) images in sea areas of the image domain. This proposed method has a prominent sidelobe suppression effect with resolution maintenance and without destruction of amplitude and phase information. This method can also be applied to SAR images of other satellites. In addition to the employment of peak sidelobe ratio (PSLR) and integrated sidelobe ratio (ISLR) in evaluating sidelobe suppression level, AE (amplitude error) and PE (phase error) are firstly defined for the evaluation of amplitude and phase-preserving quality, respectively. Through the proposed method, AE and PE values are nearly unchanged and the PSLR and ISLR are significantly reduced. The method, as an important part of the quality-improvement project of GF-3, has been successfully applied to the sidelobe suppression of GF-3 data.

## 1. Introduction

Synthetic aperture radar (SAR) is a kind of remote sensing device that can image ground targets with a high resolution. Its advantage is its ability to continuously observe targets through all weather and time. SAR is applied to many aspects, such as military and civil aspects. Until now, the most popular methods of SAR imaging are based on matched filter (MF) theory [1], such as the Range-Doppler algorithm [2], Chirp-Scaling algorithm [3], etc. These algorithms are mostly operated by Fourier transform to convert raw data to a frequency domain and are followed by matched filtering. However, these traditional SAR imaging methods based on MF have the obvious disadvantage of severe sidelobe interference. A SAR image is ideally equivalent to the approximate convolution of a scattering coefficient of the observation scene and a two-dimensional sinc function, where the resolution and sidelobe level is determined by the two-dimensional sinc function.

Under circumstances of SAR imaging without any additional sidelobe suppression, the sidelobe interference of a SAR image is obvious, this may result in several distinct disadvantages: (1) False alarm problem: there is a great possibility that the severe sidelobe of a strong scattering target is high and is mistaken as the mainlobe of a real target where a target does not exist actually. (2) Missed detection problem: The amplitude of one target is much higher than the other, in this case the low mainlobe of the weak target close to the strong target is likely to be submerged in the high sidelobe of the strong target. Sidelobe suppression of SAR images can be beneficial for target recognition. For the recently-launched Chinese GF-3 satellite, the problem of severe sidelobe interference, which reduces the quality of SAR images, also exists.

With the aim of overcoming severe sidelobe interference caused by MF-based methods, the windowing method was often adopted; however, it ran the risk of broadening the mainlobe, which could have led to a reduction of resolution in return. Thus, the nonlinear weighting method [4,5,6] was introduced later. In this way, the disadvantage of resolution reduction was conquered. In Reference [4], the method of adaptive FIR filtering was proposed and operated to control the sidelobe. Meanwhile, a non-linear apodization method was presented in Reference [5] and an analytic non-linear approach was subsequently put forward in Reference [6], through filtering and weighting. However, it was concluded that the implementation of these non-linear methods were particularly complex. In addition, the method of Spatially Variant Apodization (SVA) [7] was also used for sidelobe suppression of SAR images. However, the complicated SVA method is time-consuming and will change the scattering characteristics of targets. The major problem of severe sidelobe interference has not yet been well studied and solved, and therefore it is researched in this paper. It is important to propose a new method for sidelobe suppression. Meanwhile, the basic information of SAR images, such as the phase and amplitude of targets, needs to be maintained as well.

Nowadays, there is a promising trend in utilizing sparse reconstruction techniques for the improvement of SAR imaging quality. One of the major applications of sparse reconstruction in SAR imagery is focused on the area of image denoising [8,9,10]. In Reference [8], the method of signal denoising through sparse reconstruction was promoted, in which a unitary dictionary was applied for sparse representation. Furthermore, much attention has been paid to the field of super-resolution of SAR imaging [11,12,13], which is another major application of sparse reconstruction. All of the above applications are based upon the sparse prior of a SAR image. In the process of using these methods for super-resolution imaging, where the sidelobe suppression effect was light, it was seldom referred to. Up to now, sidelobe suppression without reducing resolution has been paid little attention.

In some special circumstances, such as SAR images of ship detection in sea areas, the main scattering targets can be considered as distributed in a sparse way over the observation scene. It is obvious that the number of dominant scatterers is much smaller than the number of overall samples in the observation scene. In such a situation, SAR images can be regarded as sparse. Therefore, sparse reconstruction can be applied in these circumstances. To overcome the severe sidelobe of traditional MF-based methods, a method based on sparsity constraint regularization is presented in this paper, where a log function is chosen to measure the sparsity of a signal. The remarkable advantage of this presented method is that it can significantly reduce the sidelobe and maintain the resolution, amplitude and phase information at the same time.

This paper is divided into five sections as follows. Section 2 presents the basic theory of sparse reconstruction. In Section 3 the reconstruction model of SAR imagery is analyzed, this is done by firstly proposing a method of sidelobe suppression without resolution reduction, amplitude and phase information distortions. Section 4 contains some numerically-simulated results and experiments of GF-3 SAR data. In addition to the use of PSLR and ISLR, three new indicators are proposed to evaluate the performance of the proposed method in Section 4. Finally, conclusions are drawn in Section 5.

## 2. Basic Theory of Sparse Reconstruction

The theory of sparse reconstruction has been previously discussed in References [14,15,16]. If the discrete signal $S\in R^{M}$ is sparse, it can be defined as:

(1) S=Dα

where $D\in C^{M\times N}$ (M≪N) is an over-complete dictionary. $\alpha \in R^{N}$ is the weighting coefficient. N is the total number of elements in α. The number of non-zero elements in α should be far less than N.

Equation (1) is an underdetermined problem and it has many solutions. However, it is possible to reconstruct the sparse signal via sparse reconstruction techniques when the dictionary D obeys the rule of Restricted Isometry Property (RIP) [17]. The RIP rule can be expressed as:

(2) $$(1-\delta_{k}){\left\|\alpha \right\|}_{2}^{2}\leq {\left\|D\alpha \right\|}_{2}^{2}\leq (1+\delta_{k}){\left\|\alpha \right\|}_{2}^{2}$$

where α is a vector with k non-zero coefficients, and $\delta_{k}\in (0,1)$. The smaller the value $\delta_{k}$ is, the more accurate the sparse signal S will be reconstructed.

According to classical CS theory [18,19,20], the *k*-sparse vector α can be recovered through the solution of optimal estimation of a $l_{0}$ norm problem as:

(3) $$\hat{\alpha }=\arg\min{\left\|\alpha \right\|}_{0}\;\mathrm{s.t.}\;S=D\alpha$$

where ${\left\|\alpha \right\|}_{0}$ denotes the 0-norm value of α, which represents the number of non-zero elements in α.

Nevertheless, the minimum 0-norm question is a NP-hard question, which is particularly sensitive to noise. Moreover, there is a great probability of instability, for the reason that the cost function is not convex. In addition, there is the disadvantage that 0-norm can only reflect the number of non-zero elements without indicating the location of each non-zero element. The 0-norm function is indeed not a suitable function to measure the sparsity of a signal.

There are several types of functions that are more suitable to measure the sparsity of a signal than the 0-norm function. For computational convenience, the sparsity measurement function is slightly modified near-zero in some applications, as long as its main properties are preserved. According to the previous study [21], several kinds of function families were recommended to measure the sparsity of a signal shown as follows:

(4) $$\psi (\alpha )=\frac{2}{\pi }\sum_{j=1}^{M}\tan^{-1}(\frac{\left|\alpha_{j}\right|}{p})$$

(5) $$\psi (\alpha )=\frac{2}{\pi }\sum_{j=1}^{M}\tan^{-1}(\frac{\left|{\alpha^{2}}_{j}\right|}{p})$$

(6) $$\psi (\alpha )=\sum_{j=1}^{M}\log_{a}(1+\frac{{\alpha^{2}}_{j}}{p})$$

where p is a small positive constant, a>1, and $\alpha \in R^{M}$. $\alpha_{j}$ represent the j−th element of α.

If ψ(α) reflects the sparsity of α, Equation (3) can be transmitted into an optimal estimation as:

(7) $$\tilde{\alpha }=\arg\min\psi (\alpha )\;\mathrm{s.t.}\;S=D\alpha$$

Assuming that noise is taken into consideration, Equation (7) can then be transmitted into:

(8) $$\tilde{\alpha }=\arg\min\psi (\alpha )\;\mathrm{s.t.}\;{\left\|S-D\alpha \right\|}_{2}<\varepsilon$$

where ε bounds the amount of noise in the measured data.

Moreover, combining the regularization theory [22], the optimization problem (Equation (8)) can be transformed into the following minimum objective function:

(9) $$\tilde{\alpha }=\arg\min J(\alpha ),J(\alpha )={\left\|s-D\alpha \right\|}_{2}^{2}+\lambda \psi (\alpha )$$

where λ is the weighted coefficient, which is operated to balance the value of ${\left\|s-D\alpha \right\|}_{2}^{2}$ and ψ(α).

## 3. Reconstruction Model of SAR Imaging and the Proposed Method

### 3.1. Reconstruction Model of SAR Imaging and Solution

The SAR imaging representation model can be described as:

(10) y=f+σ

where σ includes both background noise and sidelobes of a SAR image, y can be regarded as a complex signal with noise in an image domain, and f represents the scattering coefficient to be reconstructed from the original SAR image.

According to the theory of sparse reconstruction concluded above, the reconstruction of a SAR image utilizing the SAR representation model (Equation (10)) can be given by:

(11) $$\tilde{f}=\arg\min J(f),J(f)={\left\|y-f\right\|}_{2}^{2}+\lambda \psi (f)$$

where the former item ${\left\|y-f\right\|}_{2}^{2}$ represents the deviation between real measured data y and reconstructed data f, which reflects the matching degree of y and f; the latter item ψ(f) reflects the sparsity of f in image domain; and λ is defined as the regularization parameter, which is taken to balance the relative importance of the deviation and the sparsity. The SAR image representation model (Equation (10)) and sparsity constraint regularization is firstly combined to solve the sidelobe interference problem without lowering resolution.

In this paper, the third kind of function family (Equation (6)) is adopted to measure sparsity quality of a signal. Although the theory that a log function can be adopted as the sparsity measurement function is not new, for the first time it is applied in solving the sidelobe interference problem. Subsequently, the minimum problem Equation (11) can be rewritten as:

(12) $$\underset{f}{\arg\min}J(f),J(f)={\left\|y-f\right\|}_{2}^{2}+\lambda \log_{a}(1+\frac{|f{|}^{2}}{k})$$

Taking into consideration the situation that the sparse reconstruction theory is only suitable for real data, the SAR image is in complex form where both the amplitude information and the phase information are contained. As a result, Equation (12) can be transformed into the combination of the real and imaginary part, which can be given as:

(13) $$\begin{array}{ll} \underset{f}{\arg\min}J(f),J(f) & =\sum_{i}{\left\|y_{i}-f_{i}\right\|}_{2}^{2}+\lambda \sum_{i}\log_{a}(1+\frac{|f_{i}{|}^{2}}{k})\\ & =\sum_{i=1}^{m\times n}{\left({\left(f_{R}\right)}_{i}-{\left(y_{R}\right)}_{i}\right)}^{2}+{\left({\left(f_{I}\right)}_{i}-{\left(y_{I}\right)}_{i}\right)}^{2}+\lambda \log_{a}(1+\frac{\left({\left(f_{R}\right)}_{i}{}^{2}+{\left(f_{I}\right)}_{i}{}^{2}\right)}{k}) \end{array}$$

where $f_{i}$ and $y_{i}$ represent the i-th components of f and y, ${\left(f_{R}\right)}_{i}$ and ${\left(f_{I}\right)}_{i}$ are the real and imaginary part of $f_{i}$, and similarly ${\left(y_{R}\right)}_{i}$ and ${\left(y_{I}\right)}_{i}$ are the real and imaginary part of $y_{i}$. $\left(f,y\in C^{MN\times 1},{\left(f_{R}\right)}_{i},{\left(f_{I}\right)}_{i},{\left(y_{R}\right)}_{i},{\left(y_{I}\right)}_{i}\in R^{MN\times 1}\right)$.

Obviously, $J(f_{i})$ as the i-th component of J(f) can be written as:

(14) $$J(f_{i})={\left({\left(f_{R}\right)}_{i}-{\left(y_{R}\right)}_{i}\right)}^{2}+{\left({\left(f_{I}\right)}_{i}-{\left(y_{I}\right)}_{i}\right)}^{2}+\lambda \log_{a}(1+\frac{\left({\left(f_{R}\right)}_{i}{}^{2}+{\left(f_{I}\right)}_{i}{}^{2}\right)}{k})$$

where $J(f)=\sum_{i}J(f_{i})$, $J(f_{i})$ is continuous and differentiable in its definition domain. It is clear that $J(f)=\sum_{i}J(f_{i})$ and $J(f_{i})$ should have the same extreme point at the same place, so that the minimum optimization problem can be transformed into the problem of finding the minimum value of $J(f_{i})$.

As is known by all, the derivative of J(f) should be zero when Equation (14) reaches its minimum value. Therefore, it can be concluded that $\frac{\partial J(f_{i})}{\partial {\left(f_{R}\right)}_{i}}=0$ and $\frac{\partial J(f_{i})}{\partial {\left(f_{I}\right)}_{i}}=0$ at the minimum point. According to the analysis above, the solution of Equation (14) can be written as:

(15) $$\begin{array}{l} {\left(f_{R}\right)}_{i}={\left[1+\frac{\frac{\lambda }{\ln a}}{k+\left({\left(f_{R}\right)}_{i}^{2}+{\left(f_{I}\right)}_{i}^{2}\right)}\right]}^{-1}{\left(y_{R}\right)}_{i}\\ {\left(f_{I}\right)}_{i}={\left[1+\frac{\frac{\lambda }{\ln a}}{k+\left({\left(f_{R}\right)}_{i}^{2}+{\left(f_{I}\right)}_{i}^{2}\right)}\right]}^{-1}{\left(y_{I}\right)}_{i} \end{array}$$

where lna (a>1) is a positive constant, λ is the regularization parameter and k should be a small positive constant. The smaller the constant k is, the more accurate f will be reconstructed.

Making use of an iterative method, the solution can then be presented as:

(16) $$\begin{array}{l} {\left(f_{R}\right)}_{i}{}^{\left(n+1\right)}={\left[1+\frac{\frac{\lambda }{\ln a}}{k+{\left({\left(f_{R}\right)}_{i}^{\left(n\right)}\right)}^{2}+{\left({\left(f_{I}\right)}_{i}^{\left(n\right)}\right)}^{2}}\right]}^{-1}{\left(y_{R}\right)}_{i}\\ {\left(f_{I}\right)}_{i}{}^{\left(n+1\right)}={\left[1+\frac{\frac{\lambda }{\ln a}}{k+{\left({\left(f_{R}\right)}_{i}^{\left(n\right)}\right)}^{2}+{\left({\left(f_{I}\right)}_{i}^{\left(n\right)}\right)}^{2}}\right]}^{-1}{\left(y_{I}\right)}_{i} \end{array}$$

where $f_{i}{}^{\left(n\right)}$ represents the n−th iteration results, $f_{i}{}^{\left(n+1\right)}$ represents the (n+1)−th iteration results, ${\left(f_{R}\right)}_{i}{}^{\left(n+1\right)}$ represents the real part of $f_{i}{}^{\left(n+1\right)}$, ${\left(f_{I}\right)}_{i}{}^{\left(n+1\right)}$ represents the imaginary part of $f_{i}{}^{\left(n+1\right)}$, ${\left(f_{R}\right)}_{i}{}^{\left(n\right)}$ represents the real part of $f_{i}{}^{\left(n\right)}$, and ${\left(f_{I}\right)}_{i}{}^{\left(n\right)}$ represents the imaginary part of $f_{i}{}^{\left(n\right)}$. Generally, $\frac{\lambda }{\ln a}$ and k are positive constants.

### 3.2. Analysis of Sidelobe Suppression

**Figure 1:**
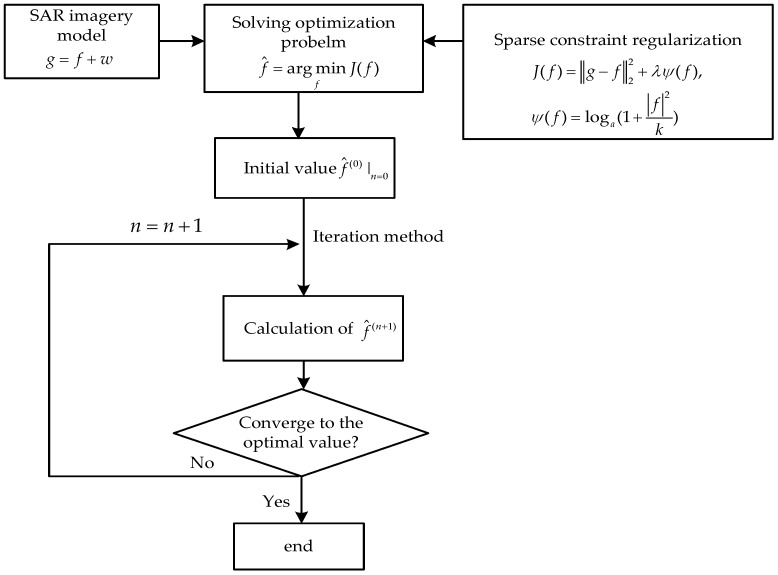
Flow chart of the presented method.

Based on the derivation presented above, the solution of sidelobe suppression can be expressed as Equation (16) and three conclusions can be drawn from it: (1) Taking the SAR image representation model Equation (10) into account, it is evident that the size and grid density of f and y stay the same as each other. Therefore, the reconstructed SAR image f should have the same resolution as the original SAR image. (2) In terms of the sparse representation theory, it has been proved that by utilizing the sparse representation technique, low sidelobe or no sidelobe could be achieved based on the selection of a proper dictionary *D* or a sparse representation model. In this paper, the dictionary *D* is the unit matrix *I*. (3) According to Equation (16), under the condition that $\frac{\lambda }{\ln a}$ and k are both a small positive constant, the value of ${\left(f_{R}\right)}_{i}$ is quite close to the value of ${\left(y_{R}\right)}_{i}$ and similarly the value of ${\left(f_{I}\right)}_{i}$ is quite close to the value of ${\left(y_{I}\right)}_{i}$; this indicates that the presented method might not destroy the amplitude and phase information of the original SAR image after sidelobe suppression processing. (4) The proposed method is processed in the image domain, so there is no need to perform computations of Fourier transform, inverse Fourier transform and complex spectral deformation in the frequency domain. All these conclusions will be supported and demonstrated below by simulations and GF-3 SAR data experiments. The main flow chart of the proposed method for sidelobe suppression with resolution maintenance, is shown in Figure 1.

## 4. Performance Assessment and Discussions

### 4.1. Evaluation Indicators of Sidelobe Suppression

In order to evaluate the performance of the proposed method, PSLR [23] and ISLR [24] are introduced to measure the sidelobe level between the original SAR image and the reconstructed SAR image through the proposed method.

To evaluate the amplitude-preserving properties of the proposed method, we first define the indicator *AE* to measure the amplitude error in the mainlobe between the original image and the reconstructed image, which can be written as:

(17) $$AE=\sqrt{\frac{\sum_{x}\sum_{y}{\left[\tilde{A}\left(x,y\right)-A\left(x,y\right)\right]}^{2}}{\sum_{x}\sum_{y}{\left[A\left(x,y\right)\right]}^{2}}}$$

where (x,y) represent coordinates of points in the mainlobe of a two-dimensional SAR image. A(x,y) and $\tilde{A}\left(x,y\right)$ denote the amplitude of point (x,y) of the original SAR image and the reconstructed one, respectively.

Similarly, *PE* is first defined as the phase error in the mainlobe between the original SAR image and the reconstructed SAR image to evaluate the phase preserving property, which can be given by:

(18) $$PE=\sqrt{\sum_{x}\sum_{y}{\left[\tilde{P}\left(x,y\right)-P\left(x,y\right)\right]}^{2}}$$

where (x,y) represent coordinates of points in the mainlobe of a two-dimensional SAR image. P(x,y) and $\tilde{P}\left(x,y\right)$ denote the phase of point (x,y) of the original SAR image and the reconstructed one, respectively.

In addition, we define *MM* to measure resolution maintenance properties between the original image and the reconstructed one, which can be given by:

(19) $$MM=\frac{Mainlobe_{recons}}{Mainlobe_{ori}}$$

where $Mainlobe_{ori}$ and $Mainlobe_{recons}$ denote the 3 dB bandwidth of the mainlobe of the original image and the reconstructed one, respectively.

### 4.2. Results and Discussion

**Figure 2:**
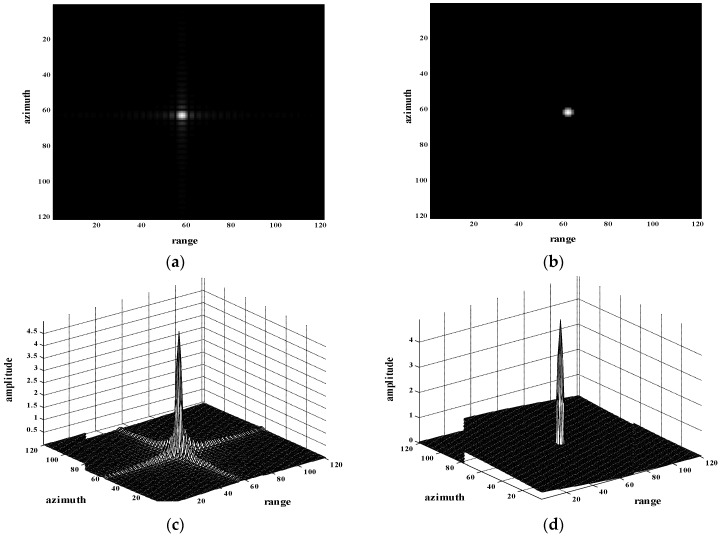
Experiment of point target (**a**) Imaging result of single-point target through conventional MF-based algorithm; (**b**) reconstructed image of (a) through the proposed method; (**c**) three-dimensional map of point target in (a); (**d**) three-dimensional map of reconstructed image in (b).

In this section, the experiment of a simulated single-point target is first presented. The images were obtained through conventional MF-based algorithm and processed with the presented method; they are displayed in Figure 2a,b. Three-dimensional maps of the original image and reconstructed image are shown in Figure 2c,d, respectively. Severe sidelobes are obvious in the original image. It is clear that the sidelobes of the reconstructed image processed by the proposed method, are far smaller than those in the original image.

**Figure 3:**
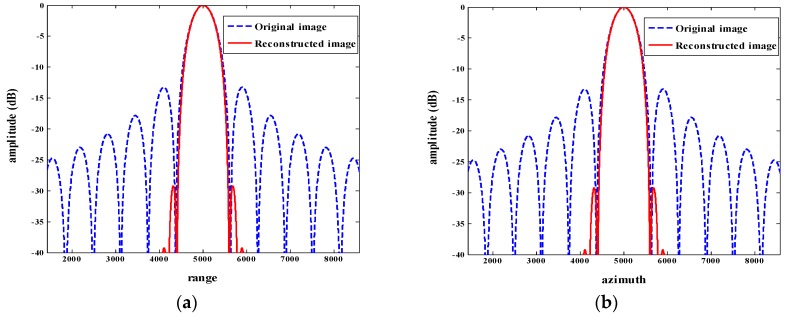
Profile map analysis of the original image and reconstructed image. (**a**) Profile map of original image and reconstructed image in range direction; (**b**) profile map of original image and reconstructed image in azimuth direction. The blue dotted line and the red solid line represent the profile map of the original image and reconstructed image, respectively.

As sketched in Figure 3, profile maps of the point target in the original image shown in Figure 2a and reconstructed image shown in Figure 2b, were performed. The width and height of the mainlobe remained almost unchanged, which proves the resolution of the reconstructed image remained unreduced in comparison with the original image. In addition, the highest sidelobe has been greatly inhibited and, at the same time, other smaller sidelobes have almost been smoothed after the processing with the proposed method.

To illustrate the performance of the proposed method in detail, values of PSLR, ISLR, AE and PE were given as follows. As shown in Table 1, the PSLR of the original image was about −13 dB in both range and azimuth directions and the PSLR of the reconstructed image reached about −29 dB, which was about 120% lower in both two directions. In Table 2, the ISLR of the original image in two directions was about −10 dB. Through processing with the proposed method, the ISLR of the reconstructed image reached about −33 dB in two directions, which was suppressed by over 200%.

Compared with the mainlobe in the original image, the mainlobe width of the reconstructed image was slightly degraded both in range and azimuth direction as shown in Table 3, which reveals that the resolution was maintained without reduction. As shown in Table 4, AE value between the original image and the reconstructed image was about 2.52%, which illustrates the slight amplitude change between the two images. Moreover, the PE value between the original image and the reconstructed one was as small as $7.27\times 10^{-33}$ radian, so that the phase change between the original image and the reconstructed one can be ignored. Overall, it can be concluded that the amplitude and phase information can be well maintained through the proposed method.

**Figure 4:**
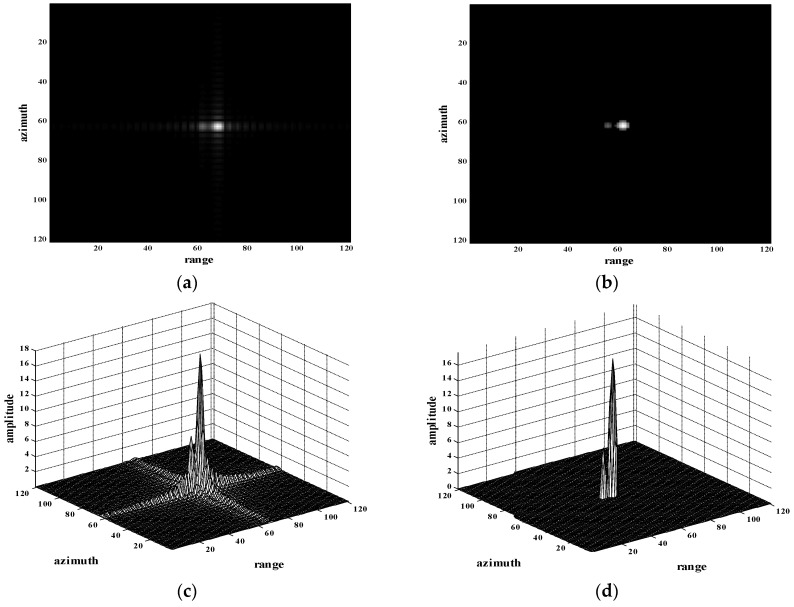
Experiment of two point targets, one of which is partly masked by sidelobes of the other (**a**) Imaging result of two point targets through a conventional MF-based algorithm; (**b**) Reconstructed image of (a) through the proposed method; (**c**) Three-dimensional map of two point targets in (a); (**d**) Three-dimensional map of the reconstructed image in (b).

In order to evaluate the performance of the proposed method further, a simulation with two point targets, one of which was partly masked by sidelobes of the other, were tested. Results are shown in Figure 4. Severe sidelobes are obvious in the original image. Two point targets are close and one of the point targets is partially submerged in the sidelobe of another strong point target, as shown in Figure 4a,c. Using the proposed method, both two point targets were well preserved with sidelobes suppressed, the results of which are shown in Figure 4b,d. The validity of the proposed method has been verified.

**Figure 5:**
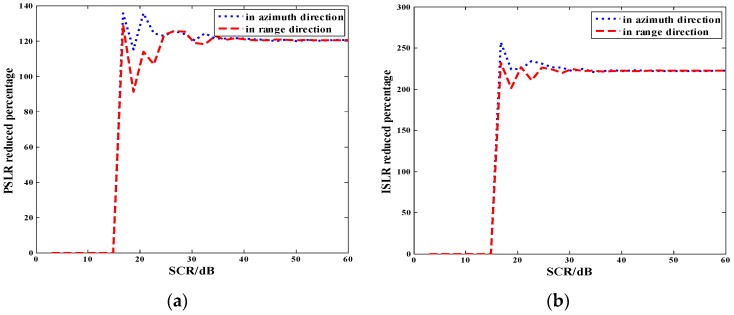
Sidelobes reduced percentage as a function of SCR for a single point target (**a**) PSLR reduced percentage for different SCRs; (**b**) ISLR reduced percentage for different SCRs.

To illustrate the sensitivity to noise of the proposed method, an analysis of the method performance as a function of signal-to-clutter ratio (SCR) was performed. Statistical results of the sidelobe reduced percentage for different SCRs from 3 dB to 60 dB, were obtained and shown in Figure 5. The sidelobes reduced percentage represents the reduced percentage of PSLR and ISLR between the SAR image, after processing with the proposed method and the unprocessed original SAR image. For every SCR, Monte Carlo simulations repeated one hundred times were applied to average the results of the sidelobes suppression effect. The proposed method was sensitive to the noise in the background. When SCR < 15 dB, the sidelobes reduced percentage was close to zero. When SCR > 15 dB, the sidelobes suppression effect was obvious. This method is suitable for SAR images with sparse targets in a relatively uniform background where SCR is greater than 15 dB. This experiment will help with defining the suitability for operational implementation of the proposed method with different SCRs.

**Figure 6:**
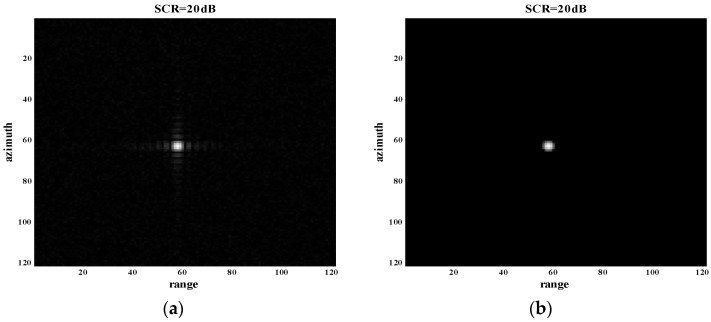
Experiment of single point target with SCR = 20 dB. (**a**) Original SAR image of single point target with SCR = 20 dB; (**b**) processed image of (a) through the proposed method.

**Figure 7:**
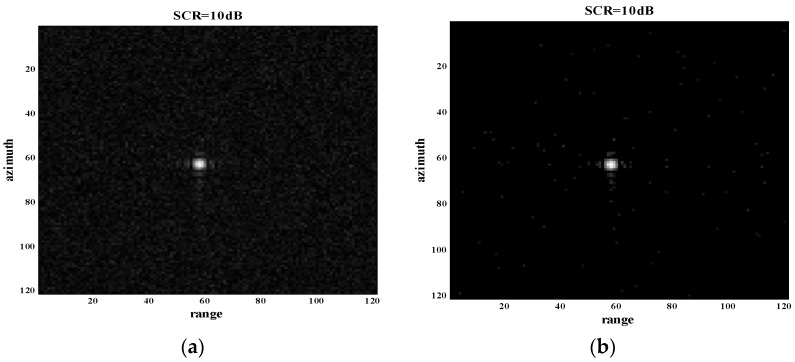
Experiment of a single point target with SCR = 10 dB. (**a**) Original SAR image of a single point target with SCR = 10 dB; (**b**) processed image of (a) through the proposed method.

Due to the sensitivity to noise in the background of the proposed method, experiments for two situations of SCR > 15 dB and SCR < 15 dB were performed in detail. Two sample cases of SCR = 20 dB and SCR = 10 dB for a single point target are shown in Figure 6 and Figure 7. From the results shown in Figure 6, we find that the proposed method was effective for sidelobe suppression when SCR = 20 dB. However, the sidelobe suppression effect was slight when SCR = 10 dB, as shown in Figure 7. It reflects the fact that the proposed method is sensitive to noise. The proposed method can be well applied to situations where SCR > 15 dB.

**Figure 8:**
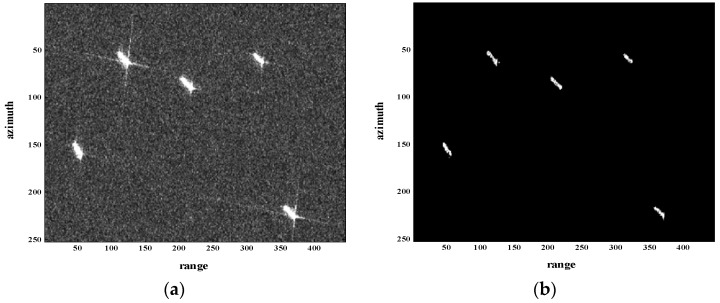
Processing result of GF-3 image around Tianjin Harbor. (**a**) GF-3 image around Tianjin harbor; (**b**) Reconstructed GF-3 image through the proposed method.

As an important part of the quality-improvement project of GF-3 satellite, GF-3 data was used extensively for testing the sidelobe suppression effect of the proposed method; some results are presented in the following. The GF-3 image around Tianjin harbor is displayed in Figure 8a. The processing result of the proposed method is shown in Figure 8b. By comparing the original GF-3 image and the reconstructed image through the proposed method, the sidelobe of ship targets has been suppressed a lot and the main details of the ship targets have been well reserved. It is concluded that the proposed method can significantly reach the goal of sidelobe suppression with resolution maintenance. However, it is worthwhile to mention that the proposed method is only suitable for scenes where targets can be regarded as sparse, especially for sea areas. For other complicated scenes, the proposed method might no longer be so effective in sidelobe suppression.

**Figure 9:**
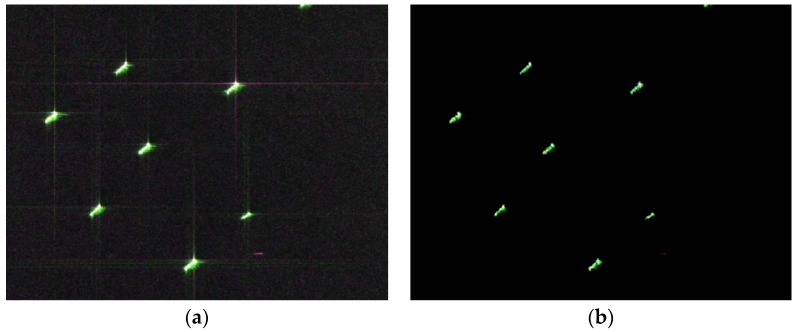
Processing results full-polarization GF-3 image of the Indian Ocean. (**a**) Full-polarization pseudo-color GF-3 image; (**b**) reconstructed full-polarization pseudo-color image of (a) through the proposed method.

A full-polarization GF-3 pseudo-color image of the Indian Ocean is displayed in Figure 9a. The pseudo-color image is formed by the fusion of HH, HV and VV polarization channel SAR images. On the one hand, sidelobes of the reconstructed image shown in Figure 9b are apparently suppressed compared with the original image. On the other hand, the color and brightness of the target remained nearly unchanged. It indicates that the proposed method does not destroy amplitude and phase characteristics. The characteristics of amplitude and phase get preserved after processing with the proposed method.

## 5. Conclusions

The purpose of this paper was to solve the severe sidelobe interference of SAR images. A new method based on sparsity constraint regularization was proposed. A log function was adopted to measure the sparsity of targets and iteration was utilized to solve the optimum problem. This method has proven to be suitable for SAR images with sparse targets in a uniform background, where the SCR is greater than 15 dB. Furthermore, to evaluate the performance of the presented method, besides the employment of PSLR and ISLR; AE, PE and MM were firstly proposed to assess the performances of amplitude, phase and resolution-preserving properties, respectively. The PSLR and ISLR were prominently reduced with maintenance of resolution, amplitude and phase information.

## Figures and Tables

**Table 1:** PSLR of the original image and reconstructed image in range and azimuth directions.

Single Point Target	PSLR in Range Direction	PSLR in Azimuth Direction
Original image	−13.2584 dB	−13.2584 dB
Reconstructed image	−29.2276 dB	−29.2276 dB
PSLR difference	−15.9691 dB	−15.9691 dB
Lowered percentage	120.45%	120.45%

**Table 2:** ISLR of the original image and reconstructed image in range and azimuth directions.

Single Point Target	ISLR in Range Direction	ISLR in Azimuth Direction
Original image	−10.2826 dB	−10.2826 dB
Reconstructed image	−33.1467 dB	−33.1467 dB
ISLR difference	−22.8641 dB	−22.8641 dB
Lowered percentage	222.36%	222.36%

**Table 3:** MM between the original image and the reconstructed image.

Parameters	Range Direction	Azimuth Direction
MM	97.84%	97.84%

**Table 4:** AE and PE between the original image and the reconstructed image.

Parameters	Point Target
AE	2.52%
PE/rad	$7.27\times 10^{-33}$

## References

[1] Cumming I.G., Wrong H.C. Digital processing of synthetic aperture radar data: Algorithms and implementation. Proceedings of the International Radar Conference.

[2] Chan Y.K., Koo V.C. (2008). An introduction to synthetic aperture radar (SAR). Prog. Electromagn. Res. B.

[3] Moreira A., Mittermayer J., Scheiber R. (1996). Extended chirp scaling algorithm for air and spaceborne SAR data processing in stripmap and ScanSAR imaging models. IEEE Trans. Geosci. Remote Sens..

[4] Degraaf S.R. (1994). Sidelobe reduction via adaptive fir filtering in SAR imagery. IEEE Trans. Image Process..

[5] Stankwitz H.C., Dallaire R.J., Fienup J.R. (1995). Nonlinear apodization for sidelobe control in SAR imagery. IEEE Trans. Aerosp. Electron. Syst..

[6] Smith B.H. (2001). An analytic nonlinear approach to sidelobe reduction. IEEE Trans. Image Process..

[7] Fischer J., Pupeza E.P.I., Scheiber R. Sidelobe suppression using SVA method for SAR images and sounding radars. Proceedings of the European Conference on Synthetic Aperture Radar (EUSAR).

[8] Protter M., Yavbeh L. (2010). Closed-form MMSE estimation for signal denoising under sparse representation modeling over a unitary dictionary. IEEE Trans. Signal Process..

[9] Moulin P., Liu J. (1999). Analysis of multiresolution image denoising schemes generalized-Gaussian priors. IEEE Trans. Inf. Theory.

[10] Aharon M., Elad M., Bruckstein A.M. (2006). The K-SVD: An algorithm for designing overcomplete dictionaries for sparse representation. IEEE Trans. Signal Process..

[11] Donoho L. (2010). Super-resolution via sparse representation. IEEE Trans. Image Process..

[12] Delaurentis M., Dickey F.M. (2002). Regularization Analysis of SAR Super-Resolution.

[13] Herman M.A., Strohmer T. (2009). High-resolution radar via compressed sensing. IEEE Trans. Signal Process..

[14] Donoho D.L. (2006). Compressed sensing. IEEE Trans. Inf. Theory.

[15] Baraniuk R.G., Cevher V., Duarte M.F. (2010). Model-based compressive sensing. IEEE Trans. Inf. Theory.

[16] Baraniuk R.G., Steeghs P. Compressive radar imaging. Proceedings of the IEEE Radar Conference.

[17] Candes E.J., Emmanuel J. (2008). The restricted isometry property and its implications for compressed sensing. C. R. Math..

[18] Candes E.J., Wakin M. (2008). An introduction to compressive sampling. IEEE Signal Process. Mag..

[19] Duarte M.F., Davenport M.A., Takhar D. (2008). Single-pixel imaging via compressive sampling. IEEE Signal Process. Mag..

[20] Tralic D., Grgic S. Signal reconstruction via compressive sensing. Proceedings of the ELMAR.

[21] Du X., Hu W., Yu W.A. (2005). A criterion for the construction of a regularization function in sparse component analysis. Circuits Syst. Signal Process..

[22] Cetin M., Karl W.C., Castanon D.A. (2000). Evaluation of a regularized SAR imaging technique based on recognition-oriented feature. Proc. SPIE.

[23] Letsch K., Berens P. (2005). PSLR estimation for SAR systems with consideration of clutter background. Proc. SPIE.

[24] Bogler P.L. (1987). Motion-compensated SAR image ISLR. IEEE Trans. Geosci. Remote Sens..

